# PMD2HD – A web tool aligning a PubMed search results page with the local German Cancer Research Centre library collection

**DOI:** 10.1186/1742-5581-2-4

**Published:** 2005-06-27

**Authors:** Andreas Bohne-Lang, Elke Lang, Anke Taube

**Affiliations:** 1 German Cancer Research Centre Heidelberg, Central Spectroscopy – Molecular Modeling, Im Neuenheimer Feld 280, D-69120 Heidelberg, Germany; 2 University of Applied Sciences Darmstadt, Information and Knowledge Management, Campus Dieburg, Max-Planck-Strasse 2, D-64807 Dieburg, Germany

## Abstract

**Background:**

Web-based searching is the accepted contemporary mode of retrieving relevant literature, and retrieving as many full text articles as possible is a typical prerequisite for research success. In most cases only a proportion of references will be directly accessible as digital reprints through displayed links. A large number of references, however, have to be verified in library catalogues and, depending on their availability, are accessible as print holdings or by interlibrary loan request.

**Methods:**

The problem of verifying local print holdings from an initial retrieval set of citations can be solved using Z39.50, an ANSI protocol for interactively querying library information systems. Numerous systems include Z39.50 interfaces and therefore can process Z39.50 interactive requests. However, the programmed query interaction command structure is non-intuitive and inaccessible to the average biomedical researcher. For the typical user, it is necessary to implement the protocol within a tool that hides and handles Z39.50 syntax, presenting a comfortable user interface.

**Results:**

PMD2HD is a web tool implementing Z39.50 to provide an appropriately functional and usable interface to integrate into the typical workflow that follows an initial PubMed literature search, providing users with an immediate asset to assist in the most tedious step in literature retrieval, checking for subscription holdings against a local online catalogue.

**Conclusion:**

PMD2HD can facilitate literature access considerably with respect to the time and cost of manual comparisons of search results with local catalogue holdings. The example presented in this article is related to the library system and collections of the German Cancer Research Centre. However, the PMD2HD software architecture and use of common Z39.50 protocol commands allow for transfer to a broad range of scientific libraries using Z39.50-compatible library information systems.

## Background

The mainstream realm of biosciences research is comprised, from the bibliographic point of view, within the accumulated indexing efforts of the United States National Library of Medicine (NLM). The PubMed [1] database currently maintained by NLM indexes 4571 journals with over 13 million articles [2] (verified on 11-Jan-2005). PubMed's web-based interface delivers comprehensive and usable access to nearly all relevant publications and even provides the option to find articles related to the initial search result. PubMed can be searched using author name(s), keywords, and other criteria as search topics. The results page displays brief information about the retrieved articles (authors, title, journal, PubMed ID) in list form. Individual resulting articles can be marked, and an individual results page can be displayed with selected articles. Opening an author link displays an abstract and typically displays the availability of an electronic full text. Electronic article availability is dependent on the searcher's institutional affiliation as expressed in the computer's Internet protocol (IP) identity. Systems like LinkOut [3] and PubMed's Outside tool [4] can provide electronic access to subscribed articles on opening the respective links article by article. Normally this selected display of relevant articles completes the task of searching PubMed.

A subsequent research working step is acquisition of relevant article full texts that lack direct linking. The individual and institutional subscription situation will provide several common alternatives, such as a local library print or e-journal holding confirmed through the local catalogue. A final resort may be a time-consuming and costly loan request.

## Methods

The web tool PMD2HD [5] has been developed to close a knowledge inequity: how can articles without full-text links embedded in PubMed be automatically interpreted for local holding status? The case for a local holding discovery tool was refined using the German Cancer Research Centre library collection. However, the tool can be adapted to use in different library systems or even for searching several collections within a network of federated libraries.

**Figure 1:**
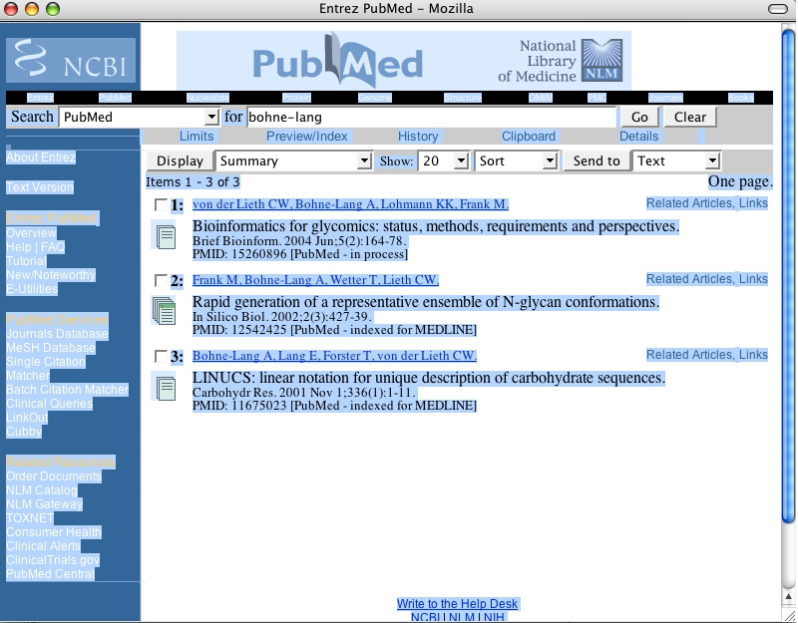
**PubMed results page. **In PubMed selected articles marked with control-a.

**Figure 2:**
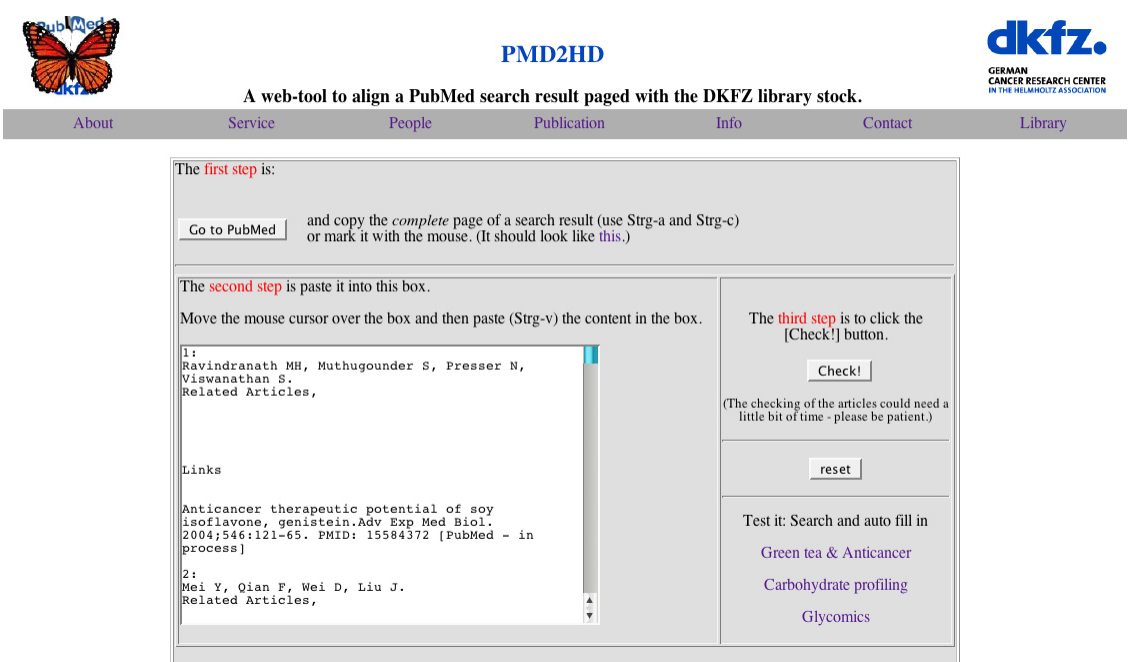
**PMD2HD web tool. **The user can paste the content from the clipboard directly into the form.

**Figure 3:**
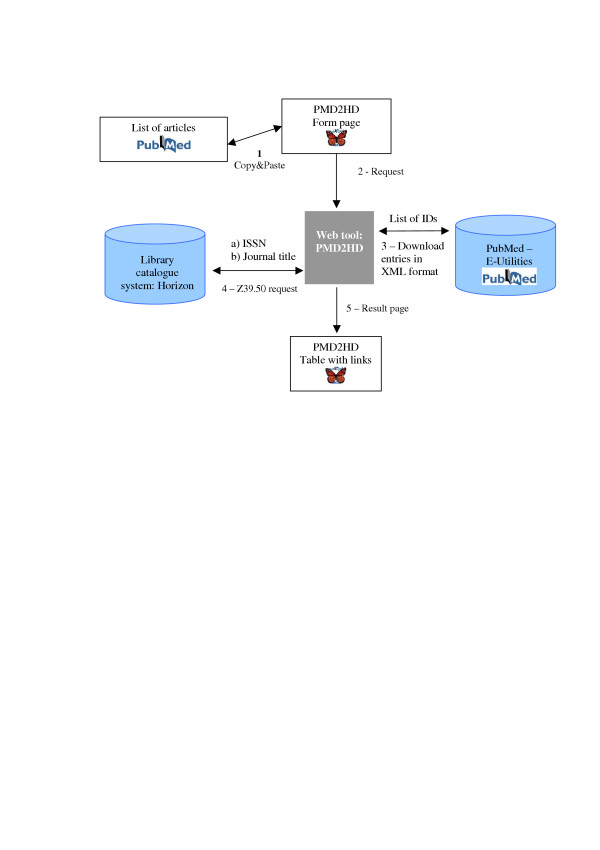
**Data processing. **The figure shows the order of the different steps. First the user selects his articles and then the data processing is triggered. The web tool asks the PubMed utilities (Entrez Programming Utilities, ) to retrieve the ISSN. With this item the Horizon system is queried. If the journal is available, the system is called again to find out if an ejournal entry is set. With all this information a results page is generated in a last step.

In the scenario envisioned for the use of PMD2HD, a researcher will can carry out a PubMed search as usual, finishing by marking (select all) the complete results web page with the shortcut command control-a (Windows™) and copying it with control-c into the clipboard (Figure 1). (This way copies the complete list to the clipboard. If the user selects only a few articles by mouse click and control-c; it works in the same way.) In the next step the complete clipboard content is pasted (control-v) into the text box of the PMD2HD tool web page (Figure 2). By pressing the [Check!] button the users trigger further data processing (Figure 3). A fundamental premise during design and development was that the tool should be as easy to use for scientists as possible, integrated into their standard workflow.

PMD2HD is written in PHP [6], a very common script language for web-based applications. The program starts with extracting all PubMed IDs (formed like: PMID: 15584372) from the entered text and arranges them in a list. Using this list the program queries the PubMed E-Utilities to retrieve a structured record in XML format [7] for each entry. Table 1 displays an example XML record. The key information of this record is the ISSN. The ISS number serves as the search argument for querying the local library system via the Z39.50 interface (at the German Cancer Research Centre, the Horizon system from Ameritech Library Services is used).

**Table 1:** XML record from PubMed. XML entry for this publication: Ravindranath MH, Muthugounder S, Presser N, Viswanathan S.Anticancer therapeutic potential of soy isoflavone, genistein. Adv Exp Med Biol. 2004; 546:121-65. PMID: 15584372

<?xml version="1.0"?>
<!DOCTYPE PubmedArticleSet PUBLIC"-//NLM//DTD PubMedArticle, 1st November 2004//EN"">
<PubmedArticleSet>
<PubmedArticle>
<MedlineCitation Owner="NLM" Status="In-Process">
<PMID>15584372</PMID>
<DateCreated>
<Year>2004</Year>
<Month>12</Month>
<Day>08</Day>
</DateCreated>
<Article PubModel="Print">
<Journal>
**<ISSN>0065-2598</ISSN>**
<JournalIssue>
<Volume>546</Volume>
<PubDate>
<Year>2004</Year>
</PubDate>
</JournalIssue>
</Journal>
<ArticleTitle>Anticancer therapeutic potential of soy isoflavone, genistein.
</ArticleTitle>
<Pagination>
<MedlinePgn>121-65</MedlinePgn>
</Pagination>
<Abstract>
<AbstractText>Genistein (4'5, 7-trihydroxyisoflavone) occurs as a glycoside...
</AbstractText>
</Abstract>
<Affiliation>Laboratory of Glycoimmunotherapy, John Wayne Cancer Institute, 2200 Santa
Monica Blvd., Santa Monica, CA 90404-2302, USA. ravi@jwci.org</Affiliation>
<AuthorList CompleteYN="Y">
<Author ValidYN="Y">
<LastName>Ravindranath</LastName>
<ForeName>Mepur H</ForeName>
<Initials>MH</Initials>
</Author>
<Author ValidYN="Y">
<LastName>Muthugounder</LastName>
<ForeName>Sakunthala</ForeName>
<Initials>S</Initials>
</Author>
<Author ValidYN="Y">
<LastName>Presser</LastName>
<ForeName>Naftali</ForeName>
<Initials>N</Initials>
</Author>
<Author ValidYN="Y">
<LastName>Viswanathan</LastName>
<ForeName>Subramanian</ForeName>
<Initials>S</Initials>
</Author>
</AuthorList>
<Language>eng</Language>
<PublicationTypeList>
<PublicationType>Journal Article</PublicationType>
</PublicationTypeList>
</Article>
<MedlineJournalInfo>
<Country>United States</Country>
**<MedlineTA>Adv Exp Med Biol</MedlineTA>**
<NlmUniqueID>0121103</NlmUniqueID>
</MedlineJournalInfo>
<CitationSubset>IM</CitationSubset>
</MedlineCitation>
<PubmedData>
<History>
<PubMedPubDate PubStatus="pubmed">
<Year>2004</Year>
<Month>12</Month>
<Day>9</Day>
<Hour>9</Hour>
<Minute>0</Minute>
</PubMedPubDate>
<PubMedPubDate PubStatus="medline">
<Year>2004</Year>
<Month>12</Month>
<Day>9</Day>
<Hour>9</Hour>
<Minute>0</Minute>
</PubMedPubDate>
</History>
<PublicationStatus>ppublish</PublicationStatus>
<ArticleIdList>
<ArticleId IdType="pubmed">15584372</ArticleId>
<ArticleId IdType="medline">101832566</ArticleId>
</ArticleIdList>
</PubmedData>
</PubmedArticle>
</PubmedArticleSet

Z39.50 [8,9] is an ANSI standard. Dating from 1996, this protocol has been specified for communication between library database systems and enables searching in heterogeneous databases. The Z39.50 protocol ensures independence from the queried catalogue database, query syntax and operating system.

If the catalogue system finds an entry for a requested ISSN, it returns all information about this entry as a record that is structured according to USMARC. One of the record items is the holding record of the journal subscription. Next, the web tool checks if the publication year of the article is in the Library's journal holding range. If so, the catalogue database is queried again via Z39.50 to get all information for this journal.

One drawback in universal applicability of this Z39.50 method is that some ejournals do not conform to an ISSN search, and occasionally a journal name lookup in the online catalogue is necessary. However, a Z39.50 journal name query is not performed by a string matching search. Instead, all names a journal has ever had, including name changes, are returned to the web tool and have to be parsed. Finally, a results page is generated showing all the data that have been collected in the two preceding steps. As PMD2HD prepares a unified list of articles with respect to both electronic and print holdings of local libraries, it can therefore be regarded as efficient tool that reduces the number of manual user actions for list collection and holding verification.

## Results

The PMD2HD results page (Figure 5) combines the main article data in a table, with a second column that describes holding and location information. Five possible actions are presented:

1. If the PubMed entry contains a DOI [10], a direct link to the full text of the article is provided. The full text can be accessed if the organization (or user) is a subscriber of this journal. This means that access is not always possible despite having found the URL of the full text.

2. The holding record of the respective print medium provided from the local library system is provided.

3. A link to the related ejournal is provided.

4. An indication 'Online?' is shown when the web tool cannot fetch an ejournal address from the online catalogue. This advises the user to check the bibliographic data manually when the stored names for the print journal and the ejournal are not exactly the same.

5. A link to the loan request web form. If the holding record of a print journal or an ejournal link is found, a link to the loan request page is not necessary.

For the link to the loan request page, the article data is filled in automatically from the retrieved data fields, and the users only have to enter their names, email addresses and local account number (Figure 5). The ISSN number automatically inserted in the comment field helps the library staff dealing with the loan request. (An in-house electronic document delivery service is planned in the future.)

## Discussion

PMD2HD is an easy-to-use web-based tool offering a usable interface and direct integration into the workflow of a typical PubMed search task. PMD2HD has also directed the Z39.50 search protocol for citation verification purposes directly at the library catalogue. PMD2HD unifies two different technical principles of data transmission, the connection-oriented Z39.50 and the non-connection client-server hypertext transmission (HTTP) data exchange. PMD2HD provides data storage that connectionless protocols cannot in terms of storing session parameters or preliminary results. Using the dedicated protocol Z39.50 overcomes the problem of inconsistencies in query languages, data format, and display styles that would ordinarily hinder a web tool's interactivity with a local library management system.

PMD2HD, with small modifications, could be further adapted for library systems with support for the Z39.50 interface, particularly focused scientific libraries and collections. In fact, the PMD2HD application could expand beyond local application to perform a cascading sequence of subsequent transactions, scalable either to a formal network of libraries or the individual needs of scientists who have access to several libraries. The common denominator requirement for alternative implementation is Z.39.50 support. Preliminary investigation in Germany demonstrated that there are two large library networks and four important libraries, including 'Die Deutsche Bibliothek', offering access via Z39.50. Examples all over the world include BIBSYS, the Library of Congress and numerous university libraries, and CURL (Consortium of University Research Libraries) in UK [11]. A test of some of the mentioned libraries showed that access was possible. An additional advantage of Z39.50 is that it does not require any commercial software investment, other than the library system already in use. Development and testing of Z39.50-based routines can be performed using YAZ, a Z39.50 client that is freely available from Index Data, Copenhagen [12].

How does PMD2HD [5] compare with existing tools such as LinkOut [3] or Outside Tool [4]? As a free PubMed service, LinkOut requires that a library uploads a list of the subscribed journal titles. The user of a certain library selects in his myNCBI the library (by using a so-called filter). Even more than one library can be selected. Now the user can perform a search, and on displaying the abstract page the icons of subscribed journals appear. PubMed's Outside Tool, in contrast to LinkOut, shows an icon that provides a link to an external program to which the PubMed ID is submitted. The program can fetch information about the article by using the PubMed ID. The service of keeping holdings up-to-date with Outside Tool is normally offered by a company, and the library has to maintain its subscription list to the company. The Outside Tool service of PubMed is free, but the service of the companies is not. Both services do not interactively check the local library system for holdings but instead rely on an uploaded list of local holdings. The PMD2HD service is independent from PubMed, yet dependent on the immediacy that a z39.50 interface to the library system provides. PMD2HD's greatest advantage is the one-page condensed listing and the inherent simplicity of that format. In contrast, both the LinkOut and Outside Tool services do not integrate the holding result of multiple records on to a single result page. The PMD2HD integrated holdings output list can be printed as a to-do list for when a library visit is necessary.

## Conclusion

Optimal benefit from PubMed literature retrieval can only be achieved when as many relevant articles as possible can be acquired in full text form as quickly as possible. PMD2HD has been created as a Web-accessible tool to perform this task on the library collection of the German Cancer Research Centre. PMD2HD helps scientists to obtain literature as fast and effortlessly as possible, and besides it saves time and money for unnecessary interlibrary loan transactions. PMD2HD can be implemented with other integrated library systems or used for retrieval within a network of federated libraries, as its underlying protocol Z39.50 is very widespread among scientific libraries. Use of Z39.50 prevents retrieval failure due to incompatibilities of query syntax and result display forms.

## List of abbreviations used

ANSI American National Standards Institute

BIBSYS A consortium for all Norwegian University Libraries, the National Library and a number of college and research libraries.

CURL Consortium of University Research Libraries

DOI Digital Object Identifier

HTTP Hypertext Transmission Protocol

ID Identification

ISSN International Standard Serial Number

IP Internet Protocol

NLM National Library of Medicine

PMID PubMed Identification

USMARC US Machine-Readable Cataloguing

XML Extensible Markup Language

## Availability and requirements

• **Project name**: PMD2HD (A web tool aligning a PubMed search results page with the DKFZ library collection.)

• **Project home page: **

• **Operating system(s): **Platform independent

• **Type of software: **Web-service

• **Programming language at server side: **PHP 4 [6]

• **Other requirements at server side: **Apache web server [11], PHP-Yaz module [12]

• **Requirements at client side: **standard web-browser

• **License: **free access

• **Any restrictions: **The access in the presented form is only useful for users of the local library.

## Competing interests

The author(s) declare that they have no competing interests.

## Authors' contributions

AL developed the web tool, EL participated in its design, co-ordination and in writing the manuscript, AT programmed the Z39.50 module with a configuration to the local library system.

**Figure 4:**
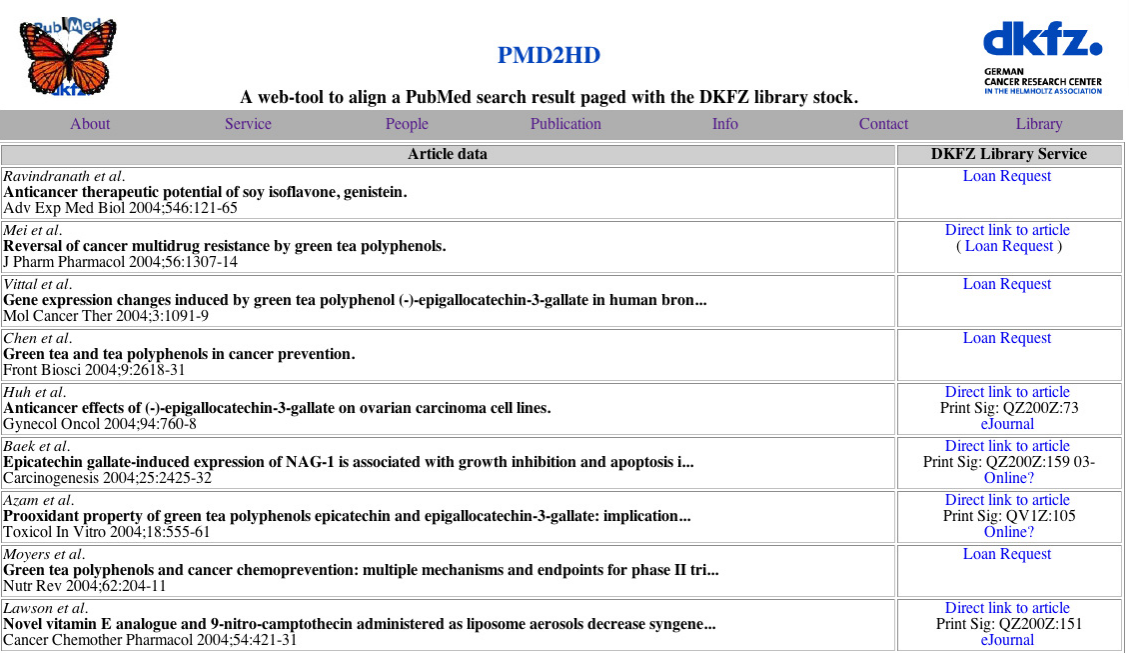
**Results page of the web tool. **The results page lists next to the article data the information on how to access it.

**Figure 5:**
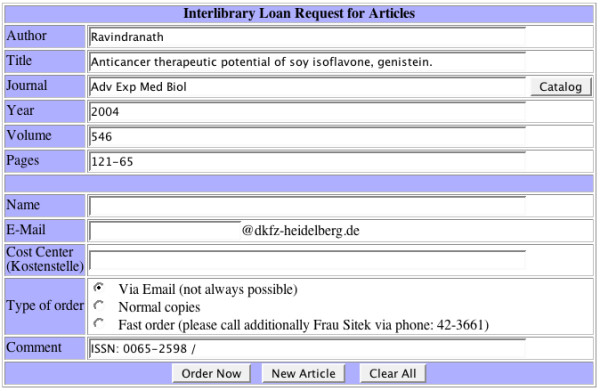
**Web form for the loan request. **The article data are filled automatically into the different form fields. The user only needs to enter his personal data like name or email.

**Figure 6:**
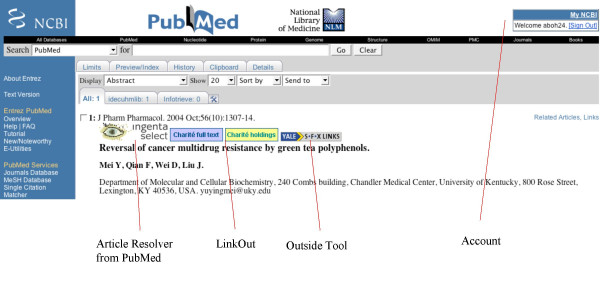
Icon gallery at PubMed abstract site with an activated LinkOut and OutSide Tool.

**Figure 7:**
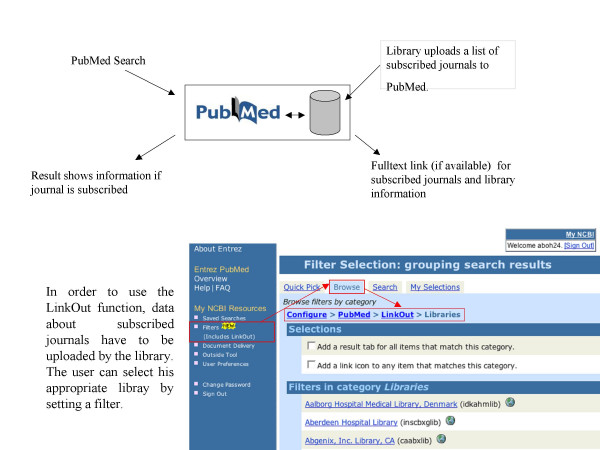
PubMed PubMed LinkOut Dataflow.

**Figure 8:**
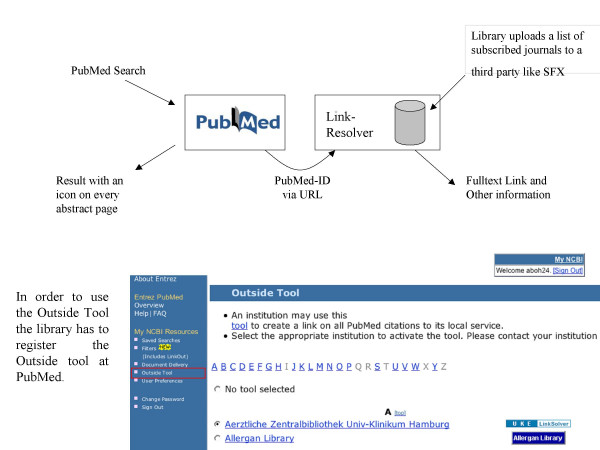
PubMed OutSide Tool Dataflow.
